# Supplementary material for: Causal Relationship between Aging and Anorexia Nervosa: A White-Matter-Microstructure-Mediated Mendelian Randomization Analysis
Source: Biomedicines. 2024 Aug 16;12(8):1874. doi: 10.3390/biomedicines12081874 (PMC11351342; doi:10.3390/biomedicines12081874)
Supplement: Supplementary file 1 [file biomedicines-12-01874-s001.zip › Supplementary Figure/Figure S8/Leaveoneout plot of MR analysis for TL to ubm-b-1624.pdf]

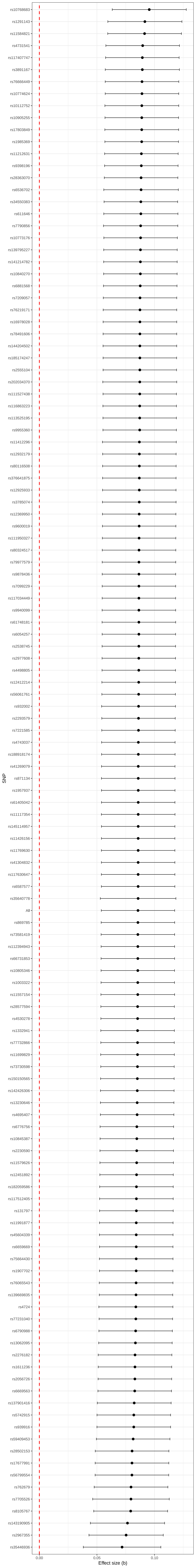
rs10768683
rs1291143
rs11584821
rs4731541
rs117407747
rs3891167
rs76666449
rs10774624
rs10112752
rs10905255
rs17803849
rs1985369
rs11212631
rs9398196
rs28363070
rs6536702
rs34550383
rs611646
rs7790856
rs10773176
rs139795227
rs141214782
rs10840270
rs6881568
rs7209057
rs76219171
rs16978028
rs78491606
rs144204502
rs185174247
rs2555104
rs202034370
rs111527438
rs116863223
rs113525195
rs9955360
rs11412296
rs12932179
rs80116508
rs376641875
rs12925933
rs3785074
rs12369950
rs9600019
rs111950327
rs80324517
rs79977579
rs9878436
rs7099229
rs117034449
rs9940099
rs61748181
rs6054257
rs2538745
rs2977608
rs4498805
rs12412214
rs56061761
rs932002
rs2293579
rs7221585
rs4743037
rs188918174
rs41269079
rs871134
rs1957937
rs61405042
rs11117354
rs145114957
rs11426156
rs11769630
rs41304832
rs117630647
rs6587577
rs35640778
All
rs869785
rs73581419
rs112394943
rs66731853
rs10805346
rs1003322
rs11557154
rs28577594
rs4530278
rs1332941
rs77732866
rs11699829
rs73730598
rs150150565
rs142426306
rs13230646
rs4695407
rs6776756
rs10845387
rs2230590
rs11579626
rs12451892
rs182059586
rs117512405
rs131797
rs11991877
rs45604339
rs6659669
rs75664430
rs1907702
rs76065543
rs139669835
rs4724
rs77231040
rs6790988
rs13062095
rs2276182
rs1611236
rs2056726
rs6669563
rs137901416
rs5742915
rs939916
rs59409453
rs28502153
rs17677991
rs56799554
rs762679
rs7705526
rs8105767
rs143190905
rs2967355
rs35446936
SNP
0.00
0.05
0.10
Effect size (b)
